# Urethro-Vaginal and Vesico-Vaginal Fistules

**Published:** 1857-11

**Authors:** N. Bozeman

**Affiliations:** Montgomery, Alabama


### Original Communications.

ART. I.—*Urethro-Vaginal and Vesico-Vaginal Fistules; Remarks upon their Peculiarities and Complications, their Classification and Treatment; Modifications of the Button Suture; Report of Cases successfully Treated.* By N. BOZEMAN, M.D., of Montgomery, Alabama.

(Continued from the July number.)

HAVING now completed, for the present, my remarks upon *Urethro-Vaginal and Vesico-Vaginal Fistules,* together with their treatment, I propose, in the next place, a brief analysis of all the cases in which I have employed the button suture. I shall then pass on to the narration of those cases which have not heretofore been recorded, at the same time accompanying each one possessing points of interest with such remarks as its practical bearing may require or circumstances suggest.

It is also proper for me to mention in this place the fact, that the foregoing remarks are merely a continuation of the views set forth in my former paper, and as such, the numbering of the cases here appended will thus appear.

My experience, although extending over a period of little more than two years, presents a greater variety of cases, I think I can safely say, than has ever before been offered to the profession by any one operator either in this country or Europe. When I say variety of cases, I refer to their different forms and complications as met with in practice, including, of course, their successful management. In making this assertion, I do not wish to be understood as boasting of my experience or success, nor do I believe any one who has examined into the subject will do me the injustice to think so. I make the statement as a fact, based upon the professional records of the past. If by it injustice is done to any one, he has only to establish his superior claims to the position I have assumed, and in that event no one would more cheerfully acknowledge the error and accord to him his just dues than myself.

The details of some of my cases are much more minute than I could have desired, but it was unavoidable under the circumstances. Their interest, and the points of practical utility they illustrate, demand, I think, that they should thus be put upon record; on these grounds, therefore, I hope to escape the imputation of prolixity.

Since the appearance of the first part of this paper, I have had under my charge two new cases, making in all twenty-six that I have now examined, instead of twenty-four as shown by the tabular statement on a former page.

Both of these were cases of single fistule, and belonged respectively to classes fourth and fifth.

One of the cases included in the statement referred to, and put down as single, was afterwards discovered to be triple, thus making three cases of this form of disease instead of two. The additional fistules in this case both belonged to the third class. These, and the two new ones, make, then, an aggregate of thirty-six fistules instead of thirty-two, which I have examined, in regard to their relative frequency of occurrence. By reference to the table the correction may be easily made. By this change in the figures, but a very slight difference in the percentage will be found to result.

The following is a summary of twenty-one cases, being all in which I have employed the *button suture.*

| | |
|---|---|
| Single fistules, . . . . . . . | 14 Cases. |
| Double " . . . . . . . . | 4 " |
| Triple " . . . . . . . . | 3 " |

| | |
|---|---|
| Class 1st, . . . . . . . . . | 5 Fistules. |
| " 2d, . . . . . . . . . | 8 " |
| " 3d, . . . . . . . . . | 6 " |
| " 4th, . . . . . . . . . | 4 " |
| " 5th, . . . . . . . . . | 8 " |

Four of the above cases, embracing seven fistules, are reported in my first paper.* Five of these fistules belonged to the second class, and two to the third.

Two more of these cases, one single and the other triple, are yet under treatment, and do not appear among the appended reports. In the former of these, the fistule belongs to the first class, and is associated with laceration of the perineum and recto-vaginal septum. It has been twice operated upon with only partial success. The failures were occasioned by a contracted and hardened condition of the surrounding parts. In the latter, the fistules (a rent and two fistules) originally belonged to the first, third, and fourth classes. Those of the first and fourth I have succeeded in permanently closing. The other was once closed, and remained so for about a week; ulcerative inflammation then caused a reproduction of it. Once since, I have operated, but failed. In all, I have had four failures. This case is remarkable, in being the one which led me to the discovery of the button suture; in being the one in which this suture (my twelfth application of it) first failed; and it is also remarkable in having been once permanently cured, and then followed by a relapse. The causes of failure may thus be briefly enumerated: bad health of the patient, almost complete closure of

* See Louisville Review, May, 1856.

the vaginal canal from old cicatrices, and its proneness to take on ulcerative inflammation whenever disturbed, either by incision, dilatation, or denudation of the edges of the fistulous openings, &c. Having succeeded in closing two of the openings permanently, and the other once, temporarily, I feel encouraged to believe that I will yet obtain a satisfactory result.

The other fifteen cases, twelve single, one double, and two triple, embracing twenty fistules, I will now proceed to relate.

CASE V. *Vesico-vaginal Fistule complicated with Rent of the Anterior Lip of the Cervix Uteri, of nearly Five Years' Standing. Cure by the Button Suture.*

Mrs. H., of Troup County, Ga., æt. 34, of medium stature, and well formed, consulted me, on the 17th of April, 1856, in regard to her condition. She states that she has had three children, two now living; the other, her last, was still-born. The birth of the latter occurred in November, 1851, at which time she became the subject of her present difficulty. Labor, in this instance, after continuing two or three days, was terminated by a resort to instruments,—craniotomy being performed. During the greater part of the labor she passed little or no urine. Says her suffering, on this account, was extreme. Just before calling in the physician who performed the above operation, she was relieved by the sudden discharge of a large quantity of water. All was now thought to be right. A few days after delivery, puerperal fever was developed, which lasted three or four weeks. At the end of this period, first called the physician's attention to the fact of the urine passing off constantly without occasioning any desire. Fistule of the bladder was now suspected. No examination, however, was made, or anything done for five or six weeks. Confinement now in the recumbent posture, with catheter worn constantly in the bladder, use of caustic, &c., constituted the course of treatment, but all to no purpose, however, as the result shows. Has never menstruated since she was injured. General health appears pretty good.

*Examination.*—Fistule of the fifth class, third variety, and complicated the anterior lip of the cervix, at the left angle of the orifice. (See description on a former page.)

*Operation.*—In the management of this particular form of injury, I heretofore stated that it is always best to make two operations. Such was the plan adopted in the present instance.

April 30th, everything being in readiness, the edges of the fistule, including the lower end of the rent, were thoroughly pared, and the sutures introduced. Of the latter, four were required. The upper one was made to take hold in the cervix on one side, and in the vesico-vaginal septum on the other, while the remaining three were all lodged in the septum, as in cases not thus complicated. Being next adjusted, the denuded parts were brought in perfect apposition. (Fig. 16 is intended to illustrate such a button as was required.)

For a few days after the operation, patient had slight fever and some little soreness across the lower part of the abdomen; otherwise got along well.

Ninth day. Removed suture apparatus, and found union of the parts perfect throughout. Catheter was worn a few days longer, and the patient then allowed to get up. Now found that she had entire control over her urine. Thus it continued for three weeks. Patient, at the end of this period, concluded she was well enough to visit a relative residing about fifteen miles in the country, and undertook the trip in a carriage. After going about two-thirds of the distance, she discovered the urine to be escaping, as it had formerly done, though in much smaller quantities. This dribbling continued until her return a few days afterwards. Upon being apprised of what had happened, I suspected at once the giving way of the newly-formed cicatrix. An examination showed such to be the case. Only the upper end of it, however, had suffered, thus causing a very small opening into the bladder to be formed. To this I attached but little importance, knowing that I should be able to reclose it without difficulty in connection with the closure of the rent next to be operated upon.

June 3d. Performed the latter operation. The sides of the cleft and the edges of the small fistule were all pared together. Three sutures were required. The lower one was introduced in the same place, and in the same manner, as the upper one in my first operation. The other two were both lodged in the substance of the cervix. Their adjustment now, and the application of a button, made to fit the inequalities of the parts, completed the operation.

After-treatment the same as usual.

Ninth day. Removed suture apparatus, and found union of the parts complete, so far as could be seen. Patient wore a catheter for four or five days longer, and then got up. Again found that she had entire control over her urine. In a few weeks she returned to her home entirely restored. Her general health now improved rapidly, and, in November following, menstruation came on for the first time since her delivery. Now it was that dribbling of urine again took place. Soon afterwards patient returned to consult me in regard to it. Stated that just before the menstrual discharge took place, she suffered severe uterine pains, and, following them, there was bloody urine and leakage of the bladder. Upon examination, no change, which the parts had undergone to allow of dribbling of the urine, could at first be discovered. When, however, the bladder was filled with water, the point at which the escape into the vagina took place was at once revealed. The water, instead of coming from a fistulous opening in the vesico-vaginal septum, was found to issue directly from the uterine orifice. A few moments' reflection convinced me as to the course the water had taken to reach this point, as well as the cause of it. After my last operation there remained at the bottom of the cleft a minute sinus, which, when the menstrual flow took place, allowed a portion of that fluid to pass down by the upper end of the cicatrix formed by the closure of the fistulous opening, and here the latter being arrested, and pressed onwards by an accumulation above, the parts had to yield, and being weaker in the direction of the bladder, the fluid very naturally took that course; hence the severe uterine pains, bloody urine, and subsequent leakage. All doubts now, as to the correctness of the above diagnosis, were removed by passing a delicate probe, slightly curved, along the track referred to in the bladder.

There remained now, I conceived, but one alternative, which was to reproduce the original cleft, by a section of the parts down to the vesical opening, then to thoroughly freshen that part of the former which had been untouched, and the sides of the latter, preparatory to the introduction of the sutures. This was done a few days afterwards, the sutures introduced, and a button applied, just as had been done in the previous operation. The result was entirely satisfactory. My patient was again discharged cured, and continues so at the time of this writing, now nearly a year since. Menstruation has been regular ever since the operation. General health perfect, &c.

*Remarks.*—This case I consider very interesting as illustrating, in a very striking manner, some of the difficulties we are liable sometimes to encounter in practice. Although a part of the original fistule was twice reproduced, yet, so far as it was concerned, I consider all of my operations as eminently successful under the circumstances. After the result of the first operation was shown, it was some three weeks before leakage of the bladder returned. This, I am satisfied, would not have taken place had my patient not attempted the journey alluded to, until the rent in the cervix could have been closed. As to the second reproduction of the fistule, the cause is to be ascribed solely to the partial failure attending the operation for closure of the rent in the cervix uteri.

The case is also interesting as affording an instance of the artificial production of *vesico-utero-vaginal fistule*, as pointed out by M. Jobert. The condition of the parts, after the second reproduction of the fistule, presented as near an approximation to the above form of injury as I have ever seen.

CASE VI. *Vesico-Vaginal Fistule complicating the Anterior Lip of the*

*Cervix Uteri, of Fourteen Years' Standing; partially closed by the Clamp Suture; Cure completed by the Button Suture.*

Mrs. H., formerly of Auburn, but now of this city, consulted me in the early part of May, 1856, in regard to her condition; æt. 46; below medium stature; stout and heavily built; general health apparently good, &c. States that in giving birth to her ninth child, June, 1842, she sustained an injury of the bladder. Since then has never been able to retain her urine. Labor lasted about thirty-six hours. The physicians in attendance, discovering that the child was dead and of large size, thought it advisable to effect speedy delivery, which they did with instruments. Says that she passed no urine during labor. Five or six days afterwards first noticed its dribbling.

Patient states that nothing was proposed for her relief until 1849, when she was advised to consult Dr. J. M. Sims, then of this city. Says that Dr. S. performed two operations upon her, from which she derived considerable benefit; the urine, after this, not dribbling off so free as it had done. Now, her general health improved, and menstruation was established. The menstrual fluid, however, instead of coming from the vagina was now discovered to be commingled, to some extent, with the urine, and, as long as the flow lasted (usually two or three days), it was attended with severe uterine pains.

*Examination.*—Fistule of fifth class, first variety; of small size, circular in shape, and situated high up, and rather to the left side. Its lower edge was unusually thin. The vaginal canal was shortened about one-third, and appeared to be a mere *cul-de-sac.* No cervix or uterine orifice was to be seen. A hard and unyielding structure, in the situation of the former, was the only remaining trace of it, and the fistule was just at the lower edge of this. In carrying a probe through the fistule it came in contact with a firm and resisting body, apparently occupying the superior fundus of the bladder, at least a part of it. This body, as I supposed, proved to be the cervix.

*Operation.*—Owing to the thinness of the border alluded to, I had great difficulty in obtaining a denuded surface sufficiently extensive to insure agglutination with the one of the opposite side. From the same cause, great trouble was experienced in the introduction of the sutures. It appeared almost impossible to prevent the needle from piercing the vesical mucous membrane. A secondary fistule, therefore, I feared from this circumstance. Three sutures were required. A few hours after the patient was removed from the operating-table, menstruation came on, attended with violent bearing-down pains. Morphia was freely given, but this served only to lull the pain, not to control it. With the cessation of the menstrual flow, the pains passed off, having lasted about three days. The remainder of the patient's confinement was unattended with anything worthy of notice.

Ninth day. Removed the suture apparatus and found complete closure of the fistule. As I feared, a small opening by the side of one of the sutures was now found to exist. The favorable appearance of this, however, induced me to think that it would close up in a few days under the use of nitrate of silver, and longer confinement in bed, with catheter in the bladder. But in this I was mistaken. At the end of four or five weeks, finding little or no improvement, I determined to employ the suture, which I did in the usual way; and in due course of time had the satisfaction of seeing the cure of the case completed. The patient now found, to her delight, that she had control over her urine—something she had been a stranger to for fourteen years. She cannot, it is true, retain her urine as long as formerly, three or four hours being as long as she can go without passing it off; still the success of the operation is perfect, and the benefit conferred by it incalculable. The inability to retain the urine longer than this, is owing, I imagine, to a diminution of the cavity of the bladder. The shortening of the vaginal canal, and incarceration of the cervix uteri, at least justify this conclusion. At the time of this writing, the patient's condition is about as it was just after the operation, excepting her general health, which has greatly improved. She continues to menstruate; being, however, irregular, as is the case generally with females who have reached her period of life. At each return, the same pains occur as formerly. The commingling of the menstrual fluid with the urine, and its entire passage through the urethra, seems to give rise to no more inconvenience than before the closure of the fistule.

*Remarks.* Whether the incarceration of the cervix uteri, in the cavity of the bladder, was the natural result of the injury which the parts sustained in parturition, or was caused by the operations which had been performed, I am not able to say. Nor am I prepared to affirm whether a part or any of it, had been destroyed. Therefore I cannot state with certainty as to what variety of my fifth class the case originally belonged. The opening I found to exist, I have said, was of the first variety, and as such, was treated in accordance with the plan heretofore laid down.

CASE VII. *Vesico-vaginal Fistule of nearly One Year's Standing, complicating the Anterior Lip of the Cervix Uteri: cure by the Button Suture.*

Amanda, colored girl, of Shelby Co., was admitted into my infirmary on the 22d of May, 1856, laboring under the above form of disease; æt. 19, stout, heavily built, and very healthy-looking.

The history of the case, as detailed to me by my friends, Drs. Wallace and Welch, who attended the patient in her accouchement, is in substance as follows: Patient was taken in labor with her first child on the 30th of Sept. 1855; pains came on regularly, and continued so until the succeeding day, when they ceased. In this condition, things continued pretty much the same for five days. Medical advice was now sought. An examination, per vaginam, showed that the head of the child was low down in the pelvis, and pressing firmly against the neck of the bladder, thus presenting an obstacle to the flow of urine, or the introduction of a catheter to relieve the organ, which was discovered to be very much distended. Relief, at this crisis, being loudly called for, and as there was no prospect of a change by the natural efforts of the patient, it was decided, at once, to employ instruments. Craniotomy was accordingly performed, and delivery speedily accomplished. No further complaint was heard from the patient until a couple of weeks had elapsed, when attention was called to the fact of dribbling of the urine, and inability to pass it in the natural way. The existence of vesico-vaginal fistule was now disclosed. In this condition patient remained until sent to me. Had not menstruated since her injury, but enjoyed pretty good health.

*Examination.*—Fistule of fifth class, first variety. (For an exact representation of its shape, size, situation, and relations, see Fig. 4.) It was an inch and a quarter in its transverse diameter.

*Operation.* May 30th, I proceeded to perform the operation in presence of the two gentlemen previously named, who visited our city for the special purpose. (See description, with form of button, Fig. 13, under its appropriate head.) Six sutures were required.

A few hours after patient was put to bed, menstruation came on; otherwise everything went on well until the night of the third day, when a severe pain in the bladder occurred, which caused the catheter to be forced out, followed by a discharge of blood. The instrument was at once replaced, and half a grain of morphia administered. No further trouble with the case was had, excepting to cleanse the catheter, and syringe out the vagina twice a day.

Ninth day. Removed suture apparatus, and found union of the parts perfect. Patient wore the catheter a few days longer, and then got up and went about as well as ever.

*Remarks.*—This case I regard as a beautiful illustration of one of the extremes of the variety to which it properly belonged. The pain referred to as causing the catheter to be forced out of the bladder, I may state here, very often occurs at this stage of the treatment. The establishment of the catamenia immediately after the operation, as in this instance, is not unusual. I have known it to take place under similar circumstances, even where it had been absent two and three years. I have several times observed this fact, and I think it is one of sufficient interest to deserve notice.

CASE VIII. *Vesico-Vaginal Fistule of Eighteen Months' Standing, complicating the Cervix Uteri with Closure of its Canal, and involving nearly the whole of the Bas Fond of the Bladder; Cure by the Button Suture.*

Minerva, colored girl, of this city, was admitted into my infirmary on the 13th of June, 1856; æt. 24; tall and rather spare built; states that she has had three children at full term, the last having been born about eighteen months ago, at which time she became the subject of her presen difficulty; is not certain how long she was in labor; thinks that it was two or three days. Child was of large size and dead, and had to be removed with instruments. Five or six days afterwards discovered dribbling of urine. Has never menstruated since; general health not been very good.

*Examination.*—Fistule of fifth class, second variety. (See Fig. 5 and its description. The drawing was made from this case.) No trace of the os uteri could be seen.

*Operation.*—July 12th, assisted by Dr. Wilson and my private pupil, Mr. J. S. Dillard, I proceeded to the operation. (See description of the different stages under its appropriate head. In addition, I may state, however, that in the paring process the end of each ureter was cut off and slit on the vaginal side of the septum to the extent of a quarter of an inch, the object of this being to throw the entrance of the urine into the bladder away from the approximated edges of the fistule.) The arrangement of the sutures, eight in number, and the distance the uterus had to be hauled down, are illustrated at Fig. 14. The button employed is illustrated by Fig. 15.

For two or three days after the operation, patient complained of considerable soreness across the lower part of the abdomen, caused doubtless by displacement of the uterus. This, however, passed off without any ill consequences.

Ninth day. Removed suture apparatus and found a perfect cure. After four or five days the catheter was dispensed with, and the patient allowed to get up. Entire control of the urine existed; could not, however, retain much at a time; had to pass it off frequently. Soon began to improve in this respect, and not long afterwards patient informed me that she could lie pretty much all night without having to get up to relieve the bladder. The general health now improved rapidly. After a few months, I undertook the re-establishment of the cervical canal. Now a more natural condition of the parts was found to exist; by the very great elasticity of the vaginal wall the uterus had been allowed to ascend almost to its normal position in the pelvis. In the situation of the uterine orifice was to be seen a very slight depression, indicating, as I thought, the entrance into the canal for which I was in search. To satisfy myself that such was the true state of things, I provided myself with a long and delicate bistoury. This I entered at the point of depression, and carried it about a quarter of an inch in the direction of the cervical canal. I next introduced a very small metallic bougie into the cut, and after some little perseverance succeeded in passing it on into the cavity of the uterus. In a short time I had the canal so far dilated that a No. 12 bougie could without any difficulty be introduced. After this I had no other trouble with the case, excepting to introduce the bougie occasionally to prevent reclosure of the orifice.

In a few weeks menstruation was re-established, and the patient's health then began to improve very rapidly. At the time of this writing, she says she is as well as she ever was. An examination of the parts now, without being apprised of their previous condition, one would, I think, be very unlikely to discover anything unusual in their appearance.

*Remarks.*—This case I regard as another fair example of the variety it represents, and in none could there be a better illustration given of the principle of the independent action of the sutures than is here shown.

The result, I need scarcely say, is truly interesting, as affording a striking instance of the practicability of closing large fistulous openings by my method, without a resort to *Cystoplasty*, thought by Jobert and others to be the only alternative in such cases.

CASE IX. *Vesico-Vaginal Fistule of Eighteen Months' Standing; Three Failures with the Clamp Suture; First Operation with the Button Suture successful.*

Ann, colored girl, of Lumpkin, Geo., was admitted on the 25th of Oct. 1856, æt. 22; below medium stature, and apparently in good health. She gives the following history of herself: States that she was confined with her first child in June, 1855; labor lasted about sixty hours; child was removed with instruments, was of large size, and dead; does not recollect whether urine was passed regularly during labor or not. First discovered dribbling five or six days after delivery.

About a year ago this patient, I understand, was placed under the charge of a surgeon, who performed three operations with the clamp suture; all to no purpose, however, as the result shows.

*Examination.*—Fistule of fourth class, first variety. (See description and the accompanying cut, Fig. 2.) The diameter in this instance was about three-fourths of an inch. Only about an eighth of an inch of the urethra seemed to be gone. No inversion of the anterior edge. Posterior edge could not be brought down so as to close the opening, nor could the vaginal canal be expanded with the speculum to the usual extent, in consequence of adhesion of the two walls of the latter upon the left side. The removal of this obstacle, therefore, was indispensable.

A few days after admission, I put the patient in position for the operation. The plan adopted consisted in making a deep and horizontal incision from the left extremity of the fistule. The result was satisfactory. The canal could now be expanded to its full size, and the posterior edge of the fistule brought in apposition with the one of the opposite side without any difficulty. Until the parts could heal, a relapse was effectually prevented by keeping the vagina constantly distended by means of an oil-silk bag of suitable size, and stuffed with bits of sponge. This tent required to be removed twice a day, and the parts syringed out with cold water.

*Operation.*—On the 16th of December, everything being in readiness for closure of the fistule, I proceeded to perform the operation in presence of Drs. Hill and Jackson. Four sutures were required. Surface upon which the button had to stand was concave; the latter had, therefore, to be adapted accordingly. (Fig. 11 is a view of such a button as was used.)

Removed suture apparatus ninth day, and found union of the parts perfect. Patient wore a catheter the usual length of time, and then got up. At first, could not retain her urine; it all passed off involuntarily through the urethra. Gave tinct. cantharides, ten drops three times a day. In a few days after commencing the medicine, there was a manifest improvement in the retentive power of the bladder, and in less than three weeks after getting up, patient was discharged entirely restored. She could now retain and pass her urine as well as she ever could.

*Remarks.*—The result in this case was as satisfactory as could have been desired; indeed more so than I had a right to expect, considering the injury which the root of the urethra had sustained. The closure of the fistule itself was simple and easy enough. When I speak of the result as satisfactory, I refer more particularly to the fact that the patient was enabled to control her urine, which, I repeat, must be regarded as remarkable under the circumstances.

The total inefficiency of the clamp suture in such cases was here clearly shown. By this I do not intend to cast any reflection upon the skill of the gentleman who employed the method in question; for I firmly believe no better result than this could have been obtained by any operator under similar circumstances.

CASE X. *Vesico-Vaginal Fistule of Thirteen Years' Standing; Inversion of the Bladder; Case thought to be Incurable; attempted Occlusion of Vagina; Failure; Fistule finally closed at One Operation by the Button Suture.*

Julia, colored girl, of Lowndes County, first came under my notice in the spring of 1853. She was at that time under the charge of Dr. J. M. Sims, then of this city. No operative procedure was attempted by that gentleman. On many occasions he remarked to me that the only alternative was occlusion of the vagina, as directed by M. Vidal. This operation, however, he never performed.

Two years afterwards, the case was placed under my charge. Its history was as follows: Patient, æt. 37; above medium size; stout, well-formed, and healthy-looking. States that at the age of twenty-five she was confined with her third child, and since then has not been able to retain her urine, not even a few drops at a time. Labor lasted four days; does not recollect whether the urine passed regularly or not during the time; child was removed with instruments; was dead, and of large size. A few days after delivery, dribbling of urine took place.

*First Examination.*—Found, in addition to the usual external signs of fistule, a very large and fleshy-looking tumor protruding from the vulva. The surface of the tumor was studded with granulations, which were firm, extremely sensitive, and would readily bleed on being handled; upon its upper part were to be seen the mouths of the ureters, from which the urine trickled away externally as fast as secreted by the kidneys. There was no difficulty, therefore, in recognizing this unnatural protrusion to be the extruded bladder. Knowing now the extensive injury which the organ must have sustained to have resulted in this condition of things, I at once came to the conclusion of Dr. Sims, namely, that occlusion of the vagina was the only alternative which promised any benefit to the patient.

A few days after admission, assisted by Dr. Clanton, I accordingly proceeded to perform that operation. A complete failure, however, was the result. I now felt but little encouraged to renew my efforts, and in view of the slight benefit which was likely to accrue to the patient from another operation, even though successful, I determined to discharge the case, hoping that I would hear no more of it. My patient, however, after being away about eighteen months, returned, insisting that I should make another attempt to relieve her in some way. In the mean time, having fallen upon the plan of the button suture, I was now prepared to take a more favorable view of the case. She was, accordingly, readmitted in December last.

*Second Examination.*—Before the true nature of the fistule could now be properly understood, or the vagina be explored, the protruded parts had to be restored to their normal place, and there retained. This reduction I succeeded very well in effecting; but to prevent their extrusion again was the trouble. For the latter purpose, the plan I adopted, and an admirable one I think it is under such circumstances, was to fill the remains of the vesical cavity with pieces of sponge. In this way, a correct and satisfactory view of the fistule and surrounding parts was, and can always be, had. The fistule was found to belong to the fourth class, third variety. (See Fig. 3, and description. The drawing was made from this case, the only one of the kind it has ever been my misfortune to meet with. The extent of inversion of the anterior border of the fistule is represented by the space between the dotted and curved lines.)

*Operation.*—On the 21st of December, assisted by Dr. Moore, I proceeded to operate, in accordance with the plan of treatment laid down in the former part of this paper.

The manner of paring the edges of the fistule and vaginal surface is intended to be shown by the circular lines delineated in the figure above referred to. The button which was employed is illustrated by Fig. 12. From this it will be seen that eight sutures were required.

Prior to commencing the operation, and for the reason before mentioned, I returned the protruded portion of the bladder, and kept it in place in the way already described. After the paring was over, and the sutures all introduced, the sponge was removed. The sutures now effectually prevented any protrusion until they could be adjusted, and then there was no likelihood that it would occur.

During the whole time of confinement the patient did well. On the ninth day, I removed the suture apparatus, and found union of the parts perfect throughout. At this result, I must confess, I was somewhat surprised. From the size of the fistule, and its peculiarities, I had my doubts about the entire success of the operation.

The patient wore a catheter five days longer, and was then allowed to get up. It was now found that dribbling of urine continued, though not to such an extent as formerly. Waiting a few days, and seeing no improvement in the retentive power of the bladder, I came to the conclusion that I was mistaken as to the entire closure of the fistule, and therefore instituted another examination. Again, however, I was satisfied that the operation had been entirely successful. The destruction of the root of the urethra, the inversion of the corresponding border of the fistule, and the peculiar manner in which the opening had been closed, sufficiently explained the incontinence under which she now suffered. I put her upon the use of tinct. cantharides, and after several weeks there was manifest improvement. She was soon able to retain her urine two or three hours in the recumbent posture, and to get up then and pass it off. In the erect posture it still could not be retained long without dribbling. In the latter respect, however, it was but a short time before there was some improvement. She could now walk about half an hour, or even longer sometimes, without incontinence, and continued gradually to improve from week to week. In conversing with her, a few days since, on the subject, she informed me that, in the morning after emptying the bladder, she could go three hours without any dribbling. This is about the extent of her improvement, and is indeed far more than could have been reasonably expected in the outset, taking all the circumstances into account.

*Remarks.*—This case, I scarcely need say, is an extreme one. The result of the operation, *per se*, is remarkable. By it is proven what may sometimes be accomplished under circumstances considered otherwise the most hopeless. By it are also established the advantages of the method adopted over any other which has heretofore been proposed in similar cases.

CASE IX.— *Vesico-Vaginal Fistule and Rent of the Urethra of Seven Years' Standing: thought to be incurable; attempted Occlusion of Vagina; Operation nearly successful; Vagina restored to its Normal State; Fistule found to be of Large Size, involving the Anterior Lip of the Cervix Uteri; both Fistule and Rent cured by the Button Suture.*

This patient was very kindly sent to me by my friend, Dr. J. S. Cummings, then of New Orleans, but now of Monroe, Louisiana. From this gentleman I learned that the case had been under treatment in New Orleans for nearly a year, and that after several unsuccessful attempts to close the fistulous opening, recourse was had to occlusion of the vagina.

*History.*—Nancy, colored girl, was admitted on the 10th of January last; æt. 27; of medium stature, stout, heavily built, and healthy-looking; states that she was confined with her third child seven years ago (1850), at which time she lost the power of controlling her water; was in labor five days; delivered by a physician; no instruments were used; five or six days afterwards first noticed the dribbling of urine. Since the injury she has menstruated, though not regularly. Health generally pretty good.

*Examination.*—Found the vagina almost completely occluded. Just beneath the meatus urinarius was a small opening into the vaginal canal. Not being able to get a view of the parts beyond through this small opening, I determined at once to restore the canal to its natural condition by severing the existing adhesions. This was quickly done, and gave rise to but little pain.

The fistule thus brought into sight was found to belong to the fifth class, first variety. In appearance it differed little, if at all, from Case VII. The rent of the urethra was about three-quarters of an inch in length. (See remarks upon this form of urethral injuries under the division of Class 1st.)

*Operation.*—Feb. 15th, the patient being in a favorable condition, I proceeded to operate, assisted by Dr. Moore. The plan was precisely the same as pointed out for Case VII. Patient did well.

On the ninth day, I removed the suture apparatus, and found perfect union, except at a small point just about the middle of the cicatrice. The cause of this slight failure was from the edge or edges of the fistule not being properly pared. The performance of this part of the operation had been very unsatisfactory, owing to the circumstance that the sky became overcast just after I commenced, and I was compelled to finish the operation, I may say, almost in the dark. I remarked at the time I feared there would be a partial failure from this cause.

Another sitting was therefore required. This was had on the 8th of March, and in due course of time a complete cure was obtained. The patient, after getting up, found that she could retain and pass her urine as well as she ever did, excepting the inconvenience resulting from the urethral injury. The rent of the urethra next claimed attention, and, I may remark here, that I had operated several times before on a case of urethral injury similar to this and had failed, from inattention to the proper support of the catheter. I had employed the button suture without any regard to this important point, hence my failures. These, however, were not without profit; I was now led to see the extent of the mischief done by the weight of the catheter, and took steps to avoid it in the present operation, which proved perfectly successful. The importance of my contrivance for the purpose above indicated is dwelt upon at sufficient length on a former page. (For a view of the instrument, which is nothing more than a modification of the button suture, see Fig. 9, and accompanying remarks.)

*Operation.*—May 19th, in the presence of Dr. J. C. Bachelor, of New Orleans, I proceeded to operate upon this case in accordance with the rules elsewhere laid down.

On the ninth day, I removed the suture apparatus, and found the urethral canal re-established, but lacking its usual length by about one-sixteenth of an inch. This slight shortening of the canal resulted, I am inclined to think, from the corners of the rent having been sacrificed in the operation which had been performed for obliteration of the vagina.

*Remarks.*—This case I regard as interesting, on account of the coexistence of fistule and rent of the urethra, a circumstance which, though not very unfrequent, is one which may always be viewed as complicating the treatment. The case is also interesting on account of the prompt and complete success of my operation, after several failures of other surgeons to close the fistule, and a resort to occlusion of the vagina. How many methods had previously been tried, I cannot state positively. Those of Vidal, Sims, and myself, all, I understand, had a showing.

CASE XII. *Urethro-Vaginal Fistule of Eight Years' Standing; Cure by the Button Suture.*

Mrs. S., of Arkansas, five years a widow, came to our city on the 16th of March last, for the purpose of consulting me in regard to her condition; æt. 29; of medium stature, well formed, and apparently in pretty good health. States that she was confined with her first child on the 17th of June, 1849, at which time she sustained an injury, supposed to be of the bladder, and ever since then has had incontinence of urine. Labor lasted about sixty hours; for the first thirty-six the pains were heavy, then they grew lighter, and finally ceased. This repose lasted only a few hours, the pains again came on with redoubled severity, and continued until the child was born, which was dead, and of large size. Says that the physician in attendance used instruments to aid in delivery. During labor the bladder was kept empty by the occasional introduction of a catheter. In less than twenty-four hours after delivery, discovered dribbling of urine. Since the injury she has enjoyed very good health. Menstruates regularly, and has had two living children and one miscarriage. These labors were all easy, and unattended with further difficulty as regards the retentive power of the bladder.

*Examination.*—Fistule of first class, third variety. (The drawing at Fig. 1 was taken from this case. See accompanying remarks.) The injury was found to be about three-fourths of an inch in length, and, as will be seen in the cut, was very near the neck of the bladder. Owing to this latter circumstance, the patient had no more control over her urine than had the opening been situated in the vesico-vaginal septum.

*Operation.*—The operation was performed on the 20th of March. The paring process in this case included both ends of the urethra. Four sutures were required, two upon either side of the urethra. Previous to their adjustment, a catheter was introduced into the canal to insure an accurate coaptation of its two ends. After the edges of the opening were brought together, there was found to exist along the course of the urethra a ridge, sloping off on either side, thus indicating a button of rather peculiar shape. Fig. 10 is a view of such a one as was applied.

For two or three days after the operation, the patient had a few darting pains in the region of the bladder, but they passed off without doing any mischief.

On the eighth day I removed the suture apparatus, and found union perfect. In a few days the patient got up. At first she had slight incontinence of urine, which I supposed to depend upon weakness of the parts, but in the course of eight or ten days this difficulty ceased entirely. She could now retain her urine as well as ever, and about a month after reaching our city she left it for her far-off home, perfectly delighted with the result.

CASE XIII. *Vesico-Vaginal Fistule complicated with a Total Loss of*

*the Vaginal Portion of the Cervix Uteri and Closure of the Cervical Canal, of Fourteen Years' Standing; Cure by the Button Suture.*

Jane, colored girl, of this county, was admitted into my infirmary on the 23d of April last; æt. 29; of medium stature, and rather delicately formed. States that she had her first child at the age of *thirteen.* Labor in this instance was unattended with difficulty. Eighteen months afterwards gave birth to a second child, at which time she was injured, and lost the power of retaining her urine. Labor lasted about forty-eight hours; child was removed with instruments (forceps, I suppose, as the child came alive). Five or six days after delivery, first discovered dribbling of urine. Has never menstruated since; general health not good. Every month has the usual premonitory symptoms of the menstrual flow, and is relieved only by bleeding at the nose.

*Examination.*—Fistule of fifth class, fourth variety. (See Fig. 8, and accompanying remarks.)

*Operation.*—This was performed on the 8th of May. For an account of it, see Fig. 17, and the appended remarks. The drawing there exhibited was made from the button employed in the case. Six sutures, it will be seen, were required.

For several days after the operation, patient had considerable pain across the lower part of the abdomen and some fever. The latter was of a remittent type, and yielded readily to the use of quinine. Otherwise she did well.

On the ninth day I removed the suture apparatus, and found perfect union. Patient wore a catheter the usual length of time, and then got up with power to retain her urine. After a few days, she said that she felt as well as she ever did, so far as retaining her urine was concerned. I have done nothing yet towards re-establishing the cervical canal, but shall so soon as the patient's health recruits a little more.

*Remarks.*—This case, it must be admitted, is another extreme one, and the ease and facility with which the cure was accomplished, leave no doubt in my mind as to the superiority of the method adopted over any other which could have been selected.

CASE XIV.—*Two Vesico-Vaginal Fistules and Rent of the Urethra, of Twelve Years' Standing; Several Operations previously performed with only partial relief; Cure by the Button Suture at two operations; Reproduction of the Rent two weeks afterwards from wearing a Catheter.*

Mrs. R., formerly of this State, but now of Louisiana, visited Montgomery, in April last, for the purpose of consulting me in reference to her case; æt. 32; tall, and rather spare built; states that she was confined with her first child, February, 1845, at which time she became the subject of her present affliction. Says that labor lasted about seventy-six hours; pains at first were slight; during the twenty-four hours immediately preceding delivery took ergot; child was still-born, and of large size. No instruments were employed. There was retention of urine before and after the birth of the child. Catheter was used as often as occasion required. Five or six days after delivery first noticed dribbling of urine, which was ascertained by the attending physicians to result from a slough in the vesico-vaginal septum. In July following, patient having sufficiently regained her health, set out in company with her husband to visit Philadelphia, whither they had been advised to go, in order to secure the best medical advice of the country.

Arriving in that city, she was placed under the charge of a well-known surgeon. The actual cautery was his plan of treatment. This course was for some time persisted in with only partial if any relief. Being discouraged at this result, advice was asked of another surgeon, no less distinguished and admired by the profession on account of his teachings and his skill as an operator. This latter gentleman, I understand, employed a form of suture which, at that time and since, has attracted no little attention on the grounds of its plausibility. Three applications of this suture were made, the last of which, if I am correctly informed, was supposed to be attended with entire success; but, as the result shows, such was not the case. For this mistake, however, no blame can be attached to the operator; for almost any one would have been deceived under the circumstances.

Patient states that she derived very great benefit from these operations, being able now to retain pretty much all her urine while in the recumbent posture. Although discharged without being permanently cured by the surgeon referred to, she possessed undiminished confidence in his integrity and skill, and deep gratitude for the very great relief he afforded her.

During the time she was undergoing the above treatment, she was confined with her second child. The labor was easy and unattended with further difficulty. Since then has passed through three others with like favorable results. Her present condition is as follows: Generally she can, by lying quiet, retain the principal part of her urine all night, but even to turn in bed, or to attempt to get up, causes it, at once, to pass off. When walking about she has not the slightest control over it. General health pretty good. Menstruation regular, &c.

*Examination.*—Discovered two fistules of the second class. One was situated upon the right, and the other, the left, equidistant from a point corresponding to the beginning of the urethra, and a little above a horizontal line drawn through the same, being about three-quarters of an inch apart. Both were circular in shape, but the one on the right considerably larger than the left. The former is the only one I saw at my first examination, and is the only one I supposed to exist at my first operation.

The rent of the urethra was about seven-eighths of an inch in length. (See remarks upon urethral injuries in the former part of this paper.) The fistulous opening discovered, although large enough to allow a considerable quantity of urine to pass off, yet was not sufficiently capacious to account for the rapidity with which the latter escaped when the patient assumed the erect posture. Another cause, therefore, I conceived must exist, and to none could the above result be more reasonably assigned than shortening of the urethra, occasioned by the rent alluded to. Reparation, then, of this canal, I regarded of the utmost importance, with a view to a successful issue in the case.

The course I determined now to pursue was to close both fistule and rent at one operation, and in that way curtail the time of confinement. I had once before performed the operation under similar circumstances. (Case not included in these reports.) The rent in that instance closed, but the fistule did not. The failure of the latter to close, I was at that time disposed to think, resulted from some oversight or neglect in the operation. Hence my desire to give the procedure a fair trial.

*Operation.*—April 23d. Performed the operation as above indicated. In paring the edges of the fistule, I found them very much hardened. Only two sutures and a plain button were required. For the rent, four sutures and such a button as Fig. 9 illustrates. (See Figure 9, and accompanying remarks.)

On the third day after the operation, the catheter became choked, and had to be removed and cleansed. I had some little difficulty in replacing it, but no mischief resulted. After this the patient got on pretty well. A small wire occasionally introduced served to keep the catheter open.

On the ninth day I removed both buttons and sutures. The rent I found entirely closed, the fistule only partially. A second failure now, under similar circumstances, satisfied me that this double operation would not do. My reasons for this belief have been already stated at length. (See former article.) Patient remained in bed five or six days longer, and then got up. After a few days more, she found that there was considerable improvement in her condition. She could now sit up and walk about for some length of time with but little leakage, consisting of what I supposed passed through the small fistulous opening remaining. A short time after this, I instituted another examination, and found the fistule which I had operated upon to all appearances closed. To be certain now that I was not mistaken, I filled the bladder with water,—the only sure test in cases of doubt. To my regret, however, I found that the fistule was not closed.

Not only this, but by the same test I was now led to the discovery of the other fistule alluded to in the outset of this report.

By close inspection, I could now see a line of cicatrization extending from one fistule to the other, running just across the mouth of the urethra, and doubtless the result of the partially successful operation previously mentioned. There is no doubt, in my mind now, that the original fistule extended across the vagina in the above situation, and that by the operation or operations, performed in Philadelphia, it was closed in its centre, thus leaving an opening at each extremity.

Here were two fistules now instead of one to be closed. One operation I determined to make suffice for both, and accordingly performed it on the 21st of May. Two sutures were required to each fistule,—all introduced antero-posteriorly. The shape of the button employed is illustrated by Fig. 10; but, in point of size, it was a little longer than the representation, and the perforations, instead of being uniformly distributed, were made to correspond to the sutures in each fistule. Used the ordinary metallic catheter. Patient complained of considerable soreness from it during her confinement in bed.

On the ninth day, as usual, I removed the suture apparatus, and found perfect closure of both fistules, with a reproduction of the rent of the urethra. The latter was caused by the weight of the catheter, the newly-formed cicatrix here not having become strong enough to bear it, a circumstance to which I have heretofore alluded in connection with the general management of rents of the urethra.

The patient upon now getting up found that she had lost, in a great measure, the control which she had over her urine after the first operation. Showing very conclusively how much the incontinence depended upon this urethral injury. An operation for this accident, therefore, became necessary, and was accordingly performed on the 8th of June following, the plan pursued being the same as in the first instance. On the ninth day I removed the suture apparatus and found union throughout, excepting a small point at the upper end, the most unfortunate place at which a failure could have occurred.

Patient again after getting up found that she could not retain her urine as she had done, the opening being large enough for the greater portion of the fluid to dribble off.

Another little operation will have to be performed, which I intend so soon as my patient's health recruits a little, for which purpose she is now absent in the country.

*Remarks.*—This case, from beginning to last, is certainly one of no ordinary interest. The existence of a large fistulous opening in the first place; its central closure by an operation; the two resulting fistules; the benefit conferred by said operation; the question whether the rent of the urethra was, in its production, cotemporaneous or not with the establishment of the original fistule; if so, the bearing which it had upon the incontinence of urine in relation to cause and effect; the necessity of closing this equally as imporant as that of the fistule, are all points deserving notice.

As to the rent, I cannot say positively whether it was produced at the same time with the fistule or not. The patient herself thinks it was. She bases her belief upon the fact, that all her subsequent labors have been easy and unattended with difficulty, and that she has never experienced any change in the power to retain her urine, since undergoing the first course of treatment.

It is true, this case is not permanently cured, yet I have no doubt of ultimate success. And although the rent of the urethra was reproduced at my second operation, and another had to be performed, which proved to be a partial failure, still the claim to having closed both the rent and the two fistulous openings, at two operations, cannot be controverted, and is an achievement of which any operator might well be proud.

CASE XI. *Three Vesico-Vaginal Fistules; One complicated with partial Loss of the Cervix Uteri, and requiring two Operations, a small Opening remaining after the second Operation, and the two other Fistules, all successfully closed at once under the same Button, followed by a secondary Fistule.*

Jane, colored girl, of West Point, Georgia, was admitted on the 11th of May last; aged about 25, and quite fleshy; has had four children, the last being born in April, 1855, at which time she sustained her present injuries; says that labor lasted four days; child was still-born and of large size. No instruments were used in effecting delivery; during labor, passed urine regularly; five days after its completion discovered dribbling, which has continued ever since; general health good; menstruation regular, &c.

*Examination.*—Three fistules were discovered (although not all at the first sitting), one belonging to the fifth class, fourth variety, and the other two to the third class. (Of the first, Fig. 7 is a representation. The uterine orifice A, owing to the anteversion of the cervix, is thrown into the cavity of the bladder.) The other two fistules were quite small, and situated opposite the uterine orifice, upon the left side, and about 3-16ths of an inch from the corresponding angle of the large fistule. They were close together, one being just above the other. Owing to their diminutive size, they were not at first discovered, and consequently were not, in the outset, taken into account in planning the course of treatment thought to be best suited to the case.

*First Operation.*—From the division of the fistule indicated in the figure alluded to, it will be evident that two operations were required. The first, for closure of the longitudinal portion, I performed May 5th, in presence of Drs. Semple, Fowler, and Foster. Four sutures only were used.

On the ninth day I removed the suture apparatus, and found union of the parts perfect.

*Second Operation*, June 18th.—Having now allowed my patient sufficient time to regain her strength, I proceeded, in the presence of Dr. Weatherby, to perform the second operation indicated, and had no little trouble in the completion of its different stages. In the first place, the situation of the cervix in the cavity of the bladder, and the smallness of the opening, were two obstacles which rendered it quite impossible to denude a surface sufficiently extensive, anterior to the uterine orifice, to insure agglutination with the edge of the opposite side, an attainment absolutely demanded, in order to get the power of bringing the os uteri into the vagina, when the fistule was closed. Not only so, but as things stood, introduction of the sutures in front of the denuded surface alluded to, would have been entirely impracticable, and the height of folly even to have attempted it. I had, therefore, to enlarge the fistule, at each extremity, to the extent of nearly half an inch. This being done, I had but little more trouble in completing the operation in accordance with the plan of treatment already laid down. Six sutures were required. These, upon being adjusted, brought the denuded surfaces in perfect apposition, and forced the os uteri to the position I intended it should occupy.

Instead now of having a plane surface for the button to rest upon, as would have naturally been expected under the circumstances, a concave one presented itself. A button, therefore, bent upon its convexity was called for, being different from the one suggested in the general plan of treatment for this variety of case. (Fig. 12 illustrates the shape of such a one as was used.)

Nothing peculiar was observed in the after-treatment.

On the ninth day I removed the suture apparatus, and found union perfect, with the uterine orifice still in its normal position.

The patient, after wearing the catheter four or five days longer, got up. Found now that there was still some leakage. After waiting several days, and learning that there was no improvement in the retentive power of the bladder, I was induced to make an examination, to see if I had not been mistaken in supposing the fistule to be entirely closed. The cicatrix, however, I found as complete as could be. The leakage was now believed to arise from another cause, and in view of that I extended my inquiry a little further in search of it. Now it was that the existence of the two small fistules, described upon the left side of the large one, were discovered, which at once accounted for the difficulty. A few days after, while examining these small openings with a view to closing both under one button, I discovered just beneath them, in the line of cicatrization formed by my second operation, what appeared to be a vesicle. This I touched lightly with nit. silver, hoping that nothing of serious import would accrue from it. An examination, however, a few days later, showed that a small fistule had formed at that point, and that urine was escaping through it. With this three fistules now existed; all of them small, and what was still more favorable, upon a line with one another, ranging obliquely upwards and to the left. The distance between the lower and middle ones was about three-fourths of an inch, and between the latter and upper about one-fourth of an inch. They could not, I conceived, have been placed in a more favorable relation to each other to justify the attempt to close all three of them at once under the same button. Having on a previous occasion succeeded in closing two openings in a similar way, I was the more readily induced to hazard an effort in this instance.

*Third Operation.*—July 28th, assisted by my partner, Dr. J. B. Gaston, I proceeded to perform the operation above intimated. The edges of all three of the openings were pared separately. The lower one required two sutures, the second one, and the third two. All being arranged, the surface was found to indicate a button not only bent upon its convexity but slightly twisted, thus giving the edge a slightly sigmoidal appearance. This contrivance I found admirably suited to the condition of the parts.

After-treatment unattended with difficulty.

On the ninth day I removed the suture apparatus, and was pleased to find complete closure of all the openings. From the lower suture, however, had resulted a secondary fistule. Being very small, I touched it with nit. silver, hoping that it would close up in a few days, which it did.

Patient now had entire control over her urine. About a week afterwards, while examining the condition of the parts, I discovered a point quite red, just in the situation of the secondary fistule, which was supposed to be closed. This I touched lightly with caustic. Very soon leakage was found to have taken place again, and an examination disclosed the fact of the existence of a small opening at that point.

Several weeks have now elapsed, and the dribbling continues. Another operation, I am satisfied, will be required for its closure. So soon as my patient regains her health—being quite feeble at this time—I shall perform it, and thus, I hope, complete the cure of the case.

*Remarks.*—This case, I need scarcely say, was much complicated, and its management tedious. The result, thus far, has indeed been more satisfactory than I had a right to expect under the circumstances. The closure of the large fistule at two operations was a remarkable result. The relapse which occurred a week or ten days afterwards was unavoidable. The closure then, at one operation, of the resulting opening, and the two others which were discovered about this time, is unequalled in the whole course of my practice. Here, then, was accomplished by three operations what under ordinary circumstances would have required five, supposing the latter to have been all successful.

The secondary fistule, which is yet unclosed, was an accident, which, in spite of every precaution, is sometimes liable to occur. This, of course, can have no bearing upon the result of the other operations.

The change of the os uteri from the cavity of the bladder to its normal position in the vagina, is a feature in this case which greatly adds to its general interest, and may well claim attention on account of the novelty of the procedure adopted.

CASE XVI. *Vesico-Vaginal Fistule complicated with Narrowing of the Vaginal Canal, of Six Months' Standing; Cure by the Button Suture.*

Mrs. L., of Crawford Co., Georgia, visited Montgomery on the 27th of last April, for the purpose of consulting me in relation to her case; æt. eighteen, of small stature, and delicately formed. Her general health is very much impaired, and she is exceedingly nervous. States that she was confined with her first child Nov. 7th, 1856. Pains for the first twenty-four hours were slight, but these became very hard, and thus continued for about twenty hours, accompanied towards the last by puerperal convulsions. No instruments were used to effect delivery. Child was still-born, and of large size. Does not know whether urine was passed regularly during labor. Her husband says that no catheter was used by the attending physician. After delivery, consciousness is said not to have returned for three days. Soon after this, first noticed dribbling of urine, which has continued unabated to the present time. Did not get out of bed for two months after confinement. Menstruation then came on, and has continued regular ever since.

*Examination.*—Fistule of second class, oval in shape, and transverse in its longest diameter, measuring about three-fourths of an inch. On each side of it, the anterior and posterior walls of the vagina firmly adhered together, thus causing a constriction at this point, and preventing a free exploration of the organ. This obstacle, therefore, had to be overcome before any other procedure could be thought of. The plan adopted for the purpose was the same as the one pointed out for Case IX, the only difference being, that in this instance lateral sections of the constricted portion had to be made from both extremities of the fistule.

*Operation.*—May 20th, the parts being in a favorable condition, I proceeded to perform the operation for closure of the fistule. The edges were pared, and the sutures, four in number, introduced in the usual way. Instead, however, of using a plain button, such as is ordinarily required in this class of cases, I found that one convex upon its under surface was indicated, such as illustrated at Fig. 11. The reason of this was the thickened and indurated condition of the parts at the extremities of the fistule. Patient went through the after-treatment without any difficulty.

Ninth day. Removed suture apparatus, and found the parts as nicely healed as could be desired. Patient wore a catheter four days longer, and then got up with entire control over her urine. She left the city about ten days afterwards, feeling, she said, as well as she ever did.

CASE XVII. *Vesico-Vaginal Fistule complicated with Narrowing of the Vaginal Canal, of Five Years' Standing; Cure by the Button Suture.*

Rachel, colored girl, of Lowndes County, was admitted into my infirmary on the 1st of May last; aged about 22, of small size, and general health very much impaired. She gives the following account of herself: When very young had her first child; the labor was unattended with difficulty. Five years ago had her second child, at which time she became the subject of her present affliction. In this instance, labor lasted three days; the pains were severe the whole time; child was removed with instruments, dead; thinks that the urine passed off regularly during labor. A couple of days after delivery first noticed dribbling of urine.

*Examination.*—Fistule of third class; oval in shape; its longest diameter transverse, and measuring something over one inch in length. The whole vaginal canal was very much contracted at this point; indeed, so much so, that it was with difficulty the cervix uteri could be seen. This obstacle was overcome in the same manner as pointed out in Case IX.

*Operation.*—July 7th. The parts being considered in a favorable condition for closure of the fistule, I proceeded to perform the required operation in presence of Drs. Semple and James. When the sutures were adjusted, the surface upon which the button was to stand was found to be concave, produced by the same cause that existed in the preceding case. A similar button, therefore, to the one used in this case, was here called for (see Fig. 11); the only difference being that, in this instance, it was required to be a little longer, and have another perforation for an additional suture.

Ninth day. Removed suture apparatus, and found a perfect cure. After eight or ten days, patient was discharged.

CASE XVIII. *Vesico-Vaginal Fistule of Ten Years' Standing: Cure by the Button Suture.*

Ann, colored girl, of this county, was admitted on the 13th of July last; aged about 28; a little above medium stature, stout, and rather heavily built. States that she was confined with her first child in her eighteenth year. Labor lasted about six days; had no trouble in passing her urine during the time; child was stillborn, and of large size. No instruments were employed to effect delivery. Four or five days after delivery first noticed the dribbling of urine. Says that this continued for a month, and then ceased entirely. With her next child she miscarried at seven months. In this instance had labor-pains two days. Very soon after delivery noticed dribbling of urine again, which has continued uninterruptedly to the present time. Had her third child four years ago; by this, no change in her condition was produced. Says that her general health has usually been good; menstruation pretty regular, &c. Her appearance now indicates an average state of health.

*Examination.*—Fistule of fifth class, first variety, quite small, and complicating the anterior lip of the cervix uteri near its centre.

*Operation.*—This was performed on the 18th of July, in the presence of Drs. Weatherby and Gaston. (See plan pointed out under the head of treatment for the above variety of cases.) Only three sutures were required. There was nothing deserving notice in the after-treatment.

Ninth day. Removed suture apparatus, and found a complete cure the result. A couple of weeks afterwards patient went home entirely restored.

*Remarks.*—This case I regard as interesting as an instance in which the fistule healed spontaneously, and was reproduced by a subsequent labor. Such was the course of things, if the patient's statement can be credited. The circumstance is certainly very remarkable, and therefore deserves a passing notice.

CASE XIX. *Vesico-Vaginal Fistule of Nine Months' Standing: Cure by the Button Suture.*

Jane, colored girl, of Columbus, Mississippi, was admitted into my infirmary, July 31st. Aged about 28, of medium stature, stout and heavily built. States that when in her nineteenth year she gave birth to her first child. Since then has had five others, all at full term. Labor unattended with trouble in all, excepting the last, which occurred in December, 1856, at which time she became the subject of her present difficulty. Says that, in this instance, the labor lasted eight days. The child, though dead and of large size, was born in the natural way. Recollects passing her urine regularly until just before the completion of labor. Two days after delivery first noticed its dribbling. Did not get out of bed for four months. After this, general health began to improve, and very soon menstruation came on.

*Examination.*—Fistule of fourth class, first variety. In a letter to me from Dr. J. W. Hopkins, also of Columbus, it is thus accurately described: "The fistule is of large size, sufficient to admit the ends of two fingers, and occupies most of the space described as the vesical triangle, particularly including the apex, or opening of the urethra, extending rather more to the right than left side. Here (upon the right) the loss of substance by sloughing was so great that the pubic arch is barely covered, affording little chance of paring the tissues in order to secure a union of the edges of the fistule. A catheter passed through the urethra comes readily into view as it emerges at the inner end, so completely is the trigonus vesicalis destroyed.

"The vagina seems much contracted, as also does the bladder. There is as yet no excoriation of the parts, either external or internal. The edges of the fistula are indurated, and, as I found upon trial, difficult to cut." He further adds: "I have attempted an operation, although I did not have much hope of success. The result realized my apprehension."

Fig. 2 is a fair illustration of this variety of cases, such as is generally met with; but there was, in this instance, a marked deviation, as appears from the above description. The fistule, instead of being transverse in reference to its longest diameter, and directly across the beginning of the urethra, presented its long axis oblique, its left extremity resting, so to speak, upon the root of the urethra. It measured about an inch in length. But little of the septum, to the left of the mesial line, was injured. Such a peculiarity I had never before met with. Across the upper end of the opening, I may also add, there was considerable constriction of the vaginal canal. This I had to overcome by deep incisions and the use of tents, before closure of the fistule could be thought of.

*Operation.*—Sept. 7th. Everything being in readiness, I proceeded to perform the necessary operation in presence of Drs. McLester and Gaston. (Fig. 11.) Five sutures were required. The button, after being bent upon its convexity, was found not to fit the parts until it was twisted in a way similar to that described in the construction of the button employed in my third operation for Case XV. After-treatment unattended with any special interest.

Ninth day. Removed suture apparatus, and found union perfect throughout. Patient wore a catheter the usual length of time, and then got up.

At the time of this writing,—ten days since the result was known,—my patient is going about quite merry, being now able to retain her urine three or four hours, without inconvenience. In a few days more she will have as good control over it as she ever had.

*Remarks.*—The history of this case fully explains its importance, as well as the difficulties I had to encounter in the operative procedure. The result, therefore, needs no comments. It speaks for itself.

*Resumé.*—Such, then, is the character of the cases of vesico-vaginal fistule that have come under my care, and such the results of my practice. It remains now for me to take a glance at some facts relating to the cases as a whole.

By reference to their histories, it will be seen that six of the accidents took place under the age of 20; seven between 20 and 25; while only two occurred at a later period,—ages 28 and 33 respectively. Six took place at first births; two at second; four at third; one at fourth; one at sixth; and one at ninth. In nine of the cases instruments were employed to aid in delivery; in six no artificial means was resorted to. The shortest duration of labor in any one of the cases was thirty-six hours, and the longest eight days, the average being about four days. In one case, dribbling of urine was observed in twenty-four hours after the completion of labor. The average length of time, however, at which this usually takes place, is, so far as I have been able to determine, about four days. Judging from the nature of the fistulous openings in the cases where instruments had been used, and where they had not, I am forced to the conclusion that nearly, if not all of them, were the result of sloughing.

The fifteen cases, embracing, as we have seen, twenty fistules, required twenty-two operations. Case V, two; Case XI, two; Case XIV, three; Case XV, four; all the others, one each.

Twenty-four operations have been performed, and two are yet required. One of the extra operations was performed for an accident which occurred in Case V, and the other was required from the partial failure of a preceding operation in Case XI.

One of the operations yet to be performed is in Case XV, and is for a secondary fistule,—in this instance, an unavoidable accident. The other is demanded in Case XIV, on account of the partial failure of the third operation.

Only two of my operations, therefore, out of the twenty-four, can properly be regarded as failures, and these only partially so.

